# Thermodynamics and Dynamics of Supercritical Water Pseudo‐Boiling

**DOI:** 10.1002/advs.202002312

**Published:** 2020-12-16

**Authors:** Florentina Maxim, Konstantinos Karalis, Pierre Boillat, Daniel T. Banuti, Jose Ignacio Marquez Damian, Bojan Niceno, Christian Ludwig

**Affiliations:** ^1^ Laboratory for Chemical Thermodynamics “Ilie Murgulescu” Institute of Physical Chemistry Splaiul Independentei 202 Bucharest 060021 Romania; ^2^ Laboratory for Bioenergy and Catalysis (LBK) ENE Division Paul Scherrer Institute Villigen PSI 5232 Switzerland; ^3^ Institute of Geology University of Bern Baltzerstrasse 1+3 Bern 3012 Switzerland; ^4^ Electrochemistry Laboratory (LEC) ENE Division Paul Scherrer Institute Villigen PSI 5232 Switzerland; ^5^ Laboratory for Neutron Scattering and Imaging (LNS) NUM Division Paul Scherrer Institute Villigen PSI 5232 Switzerland; ^6^ Department of Mechanical Engineering The University of New Mexico MSC01 1150 Albuquerque NM 87131 USA; ^7^ Spallation Physics Group European Spallation Source Lund Sweden; ^8^ Laboratory for Scientific Computing and Modelling (LSM) NES Division Paul Scherrer Institute Villigen PSI 5232 Switzerland; ^9^ Eidgenössische Technische Hochschule Zürich (ETHZ) MAVT‐LKE Zurich 8092 Switzerland; ^10^ École Polytechnique Fédérale de Lausanne (EPFL) ENAC IIE GR‐LUD Lausanne 1015 Switzerland

**Keywords:** pseudo‐boiling, supercritical water, water dynamics, water thermodynamics, Widom line

## Abstract

Supercritical fluid pseudo‐boiling (PB), recently brought to the attention of the scientific community, is the phenomenon occurring when fluid changes its structure from liquid‐like (LL) to gas‐like (GL) states across the Widom line. This work provides the first quantitative analysis on the thermodynamics and the dynamics of water's PB, since the understanding of this phase transition is mandatory for the successful implementation of technologies using supercritical water (scH_2_O) for environmental, energy, and nanomaterial applications. The study combines computational techniques with in situ neutron imaging measurements. The results demonstrate that, during isobaric heating close to the critical point, while water density drops by a factor of three in the PB transitional region, the system needs >16 times less energy to increase its temperature by 1 K than to change its structure from LL to GL phase. Above the PB‐Widom line, the structure of LL water consists mainly of tetramers and trimers, while below the line mostly dimers and monomers form in the GL phase. At atomic level, the PB dynamics are similar to those of the subcritical water vaporization. This fundamental knowledge has great impact on water science, as it helps to establish the structure–properties relationship of scH_2_O.

## Graphical abstract

Pseudo‐boiling is a new phenomenon to be considered for the successful implementation of technologies using supercritical fluids. This experimentally supported computational work provides the first quantitative analysis on water's pseudo‐boiling. The fundamental knowledge attained in this study has great impact on water science, as it helps to establish the structure–properties relationship of water at supercritical conditions.

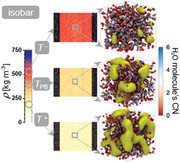

## 1 Introduction

Emerging technologies using scH_2_O as a reaction medium are promising solutions helping to achieve the 6th and the 7th Sustainable Development Goals on UN's new 2030 agenda.^[^ ^1^ ^]^ For instance, clean water and sanitation (the 6th) can be based on scH_2_O desalination^[^ ^2^ ^]^ and on wastewater treatment by scH_2_O oxidation.^[^ ^3^ ^]^ Moreover, clean and affordable energy (the 7th) can be harvested by scH_2_O gasification of wet biomass.^[^ ^4^ ^]^ Although considered green and energetically effective, supercritical water technologies are still not implemented at large scale, mainly due to harsh operating conditions implying high costs. Optimizing the process parameters, such as temperature, pressure, residence/reaction time, as well as using catalysts can lower the costs. For this, managing to finely tune the properties of water is of great interest, especially near the critical point (*T*_cr_, *p*_cr_, and *ρ*_cr_), where the thermodynamic, transport, and solvent properties of water show drastic changes. For instance, at only few bars above the critical pressure of water, in a temperature interval of a few *K*, the density drops from 600 to 150 kg m^−3^, the specific enthalpy increases ≈400 J kg^−1^, the self‐diffusion coefficient increases by a factor of 2, and the dielectric constant drops to almost a third. These property changes are practically accompanying the phase change from liquid‐like (LL) to gas‐like (GL) states of water.

Most frequently in the literature, there are two names associated with the transitional lines between different LL and GL regimes in the supercritical region of a fluid, and these names are Widom and Frenkel.^[^ ^5^, ^6^, ^7^, ^8^ ^]^ The transition at the Widom line (or Nishikawa's ridge^[^ ^9^, ^10^ ^]^) is purely thermodynamic, as it relates to the thermodynamic anomalies in the fluid's critical behavior. For example, the compressibility coefficient, thermal expansion coefficient, heat capacity, and the density fluctuations go through maxima upon varying pressure or temperature. Consequently, there is a whole set of “lines of maxima” of various thermodynamic quantities in the supercritical region and all these lines merge asymptotically into a single line when approaching the critical point.^[^ ^6^ ^]^ Notably, the Widom line depends on the used quantity, and differs between fluids.^[^ ^11^, ^12^, ^13^ ^]^ Across the Frenkel line, closely related to the Fisher‐Widom line,^[^ ^6^, ^12^ ^]^ the transition is dynamic and the LL and GL phases at this transition are different in terms of diffusion mechanisms.^[^ ^6^, ^14^ ^]^ More precisely, the Frenkel line demarcates two regions in which the fluid behaves as nonrigid liquid (dense gas‐like behavior) and rigid liquid (solid‐like behavior).^[^ ^15^, ^16^, ^17^ ^]^ One big difference between the Frenkel line and the Widom line is that the latter has an upper pressure limit between 3 and 10 reduced pressure, whereas the Frenkel line does not.^[^ ^18^ ^]^ In the case of scH_2_O, the Frenkel line originates at pressures higher than 380 bar.^[^ ^15^ ^]^

During isobaric heating of a fluid and at reduced pressure (*p*_r_  =  *p*/*p*_cr_) lower than 3, the LL to GL phase transition upon crossing the Widom line is referred to as supercritical pseudo‐boiling (PB),^[^ ^19^ ^]^ the phenomenon obeying similar laws as subcritical boiling^[^ ^13^ ^]^ in that it is associated with a large heat capacity (*C*_p_) and a steep change in density. Differently is that, in the supercritical region, PB is a continuous transition from LL to GL states of the fluid, happening over a finite temperature interval rather than at a constant saturation temperature as boiling does. At constant pressure, the PB transition starts at a temperature *T* ^−^, when the *C*_p_ of the fluid starts to deviate from its liquid value, then the fluid reaches the temperature of transition at *T*_PB_, when its *C*_p_ has the maximum value corresponding to the Widom line, and the PB transition ends at temperature *T* ^+^, when *C*_p_ approaches its ideal gas value.^[^ ^19^ ^]^ The most important realization of PB is that the added energy to the system is used to *both* overcome molecular attraction *and* raise the temperature of the fluid simultaneously, the first being a structural (st) and the latter a thermal (th) contribution.^[^ ^19^ ^]^ Moreover, associated with the PB transition there is a distributed latent heat.^[^ ^20^ ^]^

Although very important to be understood for the successful implementation of the scH_2_O applications, the PB phenomenon was mostly studied for fluids other than water, such as nitrogen or argon.^[^ ^21^, ^22^ ^]^ Recently our group reported on the visualization of scH_2_O PB by neutron imaging and showed that LL and GL phases of water can be distinguished experimentally at millimeters scale when water interacts with microporous carbon with a hydrophobic surface.^[^ ^23^ ^]^ Our results reported in ref. ^[^ ^23^ ^]^ open the way to better understand the PB phase transition for water. However, for the optimization of scH_2_O reactors, a quantitative analysis of the phenomenon is needed. Very recently, it was pointed out that, in order to gain insights into the impact of the process parameters under a large variety of conditions, both analyses at macroscale and at molecular scale are needed to estimate thermodynamic and transport properties as well as the kinetic parameters of reactions taking place under supercritical conditions of a fluid.^[^ ^24^ ^]^

Since the early nineties of the last century, the structure of scH_2_O has been extensively studied by both experimental methods and molecular simulations. The experimental approaches are mainly based on techniques such as neutron^[^ ^25^, ^26^, ^27^ ^]^ and X‐ray diffraction,^[^ ^28^ ^]^ IR,^[^ ^28^, ^29^ ^]^ and Raman^[^ ^30^, ^31^ ^]^ spectroscopies, and NMR.^[^ ^32^ ^]^ The simulation techniques used to analyze the structure of supercritical fluids are reviewed in ref. ^[^ ^33^ ^]^ and for water these include mostly molecular dynamics (MD) simulations^[^ ^34^, ^35^, ^36^, ^37^, ^38^, ^39^, ^40^ ^]^ (ab initio MD^[^ ^30^, ^41^ ^]^), Monte Carlo simulations^[^ ^27^, ^42^, ^43^ ^]^ (Reverse Monte Carlo^[^ ^39^ ^]^), and density functional theory.^[^ ^30^ ^]^ The above‐mentioned studies have been mainly focused on changes in the scH_2_O structure described in terms of hydrogen bonding. The first debate was on the existence of hydrogen bonds in scH_2_O. Postorino et al. in 1994 claimed, by analyzing angle‐averaged pair distribution function for O—H measured in neutron diffraction, that at a supercritical temperature of 673 K and a water density of 660 kg m^−3^ almost all hydrogen bonds are broken down.^[^ ^25^ ^]^ However, interpretation of either the first peak of the O—H intermolecular radial distribution function or its volume integral as representative of the degree of hydrogen bonding has been found to be inappropriate.^[^ ^26^ ^]^ Experimentally, it has been demonstrated by both spectroscopic and diffraction techniques that hydrogen bonds are still preserved up to 800 K in the supercritical region.^[^ ^28^ ^]^ By interpreting the chemical shift in NMR, it was demonstrated that there are still 29% as many hydrogen bonds at 673 K and 400 bar (*ρ * =  520 kg m^−3^) as for room temperature water.^[^ ^32^ ^]^ Based on a hybrid distance‐energy criterion of hydrogen bonding, it was demonstrated that at supercritical conditions of 673 K and a water density of 660 kg m^−3^, the average hydrogen bonds are almost 10% weaker, 5% longer, and about 8° more bent, compared to those in normal liquid water. However, over 40% of them are still preserved in the supercritical state.^[^ ^42^ ^]^ The second point discussed in relation to the scH_2_O structure is the change in symmetry of the hydrogen bonds network. Tetrahedral orientation of the hydrogen bonded neighbors is already lost at 423 K, whereas the hydrogen bonds themselves remain preferentially linear even above the critical point. In investigating the properties of the hydrogen‐bonded clusters of the molecules it has been found that the space‐filling percolating network, present under ambient conditions, collapses around the critical point.^[^ ^39^ ^]^ Water near and above the critical point fragments mostly into trimers, dimers, and single molecules at low densities, while at high densities more complex structures appear, which are anomalous with respect to the normal liquid phase.^[^ ^41^ ^]^ Very recently, also the machine learning approach has been proposed for understanding the physics of supercritical fluids at atomic level.^[^ ^44^ ^]^

Here, we present the experimental and theoretical evidence of the PB phenomenon related to the phase transition of water from LL to GL supercritical state. Theoretically, we analyze this thermodynamic transition, which reflects the macroscopic changes in water properties, using the concept of supercritical fluid PB at Widom line crossover.^[^ ^19^ ^]^ Experimentally, we follow the PB transition through the water density variation translated in color changes of the neutron images. More, we evaluate by MD the water's structural changes when the system goes from LL to GL phases.

## 2 Results

### 2.1 Macroscopic View of the Transition

**Figure 1:**
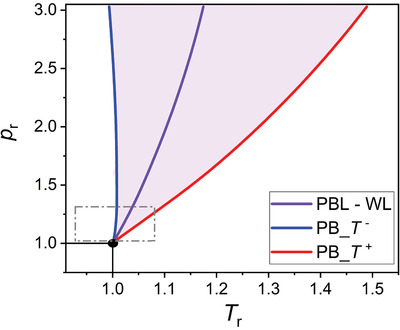
Water phase diagram in reduced pressure–reduced temperature space showing the PB transitional region (light violet); it is calculated from NIST data and based on Banuti's theory;^[^ ^19^ ^]^ at constant pressure, the PB starts at the blue line (corresponding to *T* ^−^) and ends at the red line (corresponding to *T* ^+^); the violet line is the PB–Widom line, corresponding to *T*_PB_; PB transitional region widens as the pressure increases; gray dashed square shows the region in the water phase diagram of our analysis by neutron imaging and MD.

To obtain information on the thermodynamics of the LL to GL water phase transition, we determined the PB transitional region in the water phase diagram based on Banuti's theory^[^ ^19^ ^]^ and using NIST reference data for water heat capacity^[^ ^45^ ^]^ (see the Experimental Section for details). **Figure** **1** presents the water phase diagram in the reduced temperature (*T*_r_)–reduced pressure (*p*_r_) space with the indication of the PB transitional region (light violet). The blue and the red lines are the boundaries of the PB zone, and correspond to the start (*T* ^−^) and endpoint (*T* ^+^), respectively, of the isobaric transition. The violet line is the Widom line indicating the temperature of maximum *C*_p_ for each isobar, which is considered to be the temperature of PB transition (*T*_PB_), and therefore is referred hereafter to as PB–Widom line. The widening of the PB transitional region with increasing pressure is evident. The gray dashed square in Figure 1 indicates the scH_2_O state region in the vicinity of the critical point, and analyzed in this study by neutron imaging and MD simulations.

**Figure 2:**
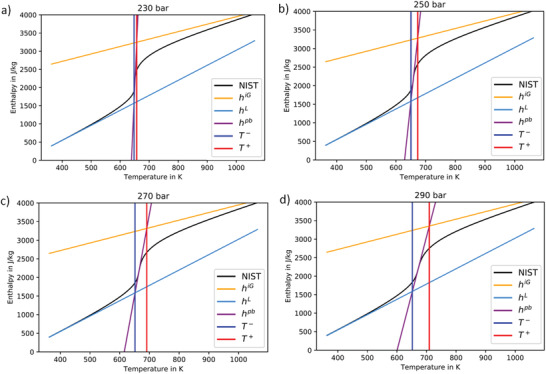
Water enthalpy variation versus temperature along different isobars; it shows the determination of PB temperature interval (*T* ^+^ – *T* ^−^) from enthalpy asymptotes for ideal gas and liquid references^[^ ^19^ ^]^ based on NIST reference data;^[^ ^45^ ^]^ the PB temperature interval and the enthalpy of transition (*h* ^pb^) increase with the pressure.

**Figure** **2** shows the variation of water enthalpy with temperature and the determination of the PB temperature interval (*T* ^+^ – *T* ^−^) for water at a) 230, b) 250, c) 270, and d) 290 bar using enthalpy asymptotes for the ideal gas (*h* ^iG^) and liquid (*h* ^L^) reference states.^[^ ^19^ ^]^ Notably, the higher the pressure, the larger the temperature interval of the phase transition. **Table** **1** presents the thermodynamic data calculated according to Banuti's theory^[^ ^19^ ^]^ for the PB transition at different pressures analyzed in this study. In the studied pressure range of 65 bars, the PB temperature increases by more than 20 K and the transition temperature interval at 290 bar is ten times larger than at 230 bar. From a practical point of view, it is of interest to evaluate the ratio between the structural and the thermal contributions of PB, which shows how much the energy required for the structural changes exceeds the thermal energy necessary to heat the fluid during PB.^[^ ^19^ ^]^ For instance, our results show that almost 39 times more energy is required for the system to change the structure from LL to GL state than to increase the temperature of water by ≈2.4 K at 225 bar during the PB phase transition (Table 1).

**Table 1:** Thermodynamic data calculated for PB transition at different pressures: *T*_PB_ is the temperature of transition, Δ_PB_ *T* is the transition temperature interval (*T* ^+^ – *T* ^−^), Δ_PB_ *h* is the enthalpy of transition, Δ*h*_st_/Δ*h*_th_ is the ratio between the structural and thermal contributions to PB, and Δ_PB_ *s* is the entropy of transition

*p* [bar]	*T*_PB_ [K]	Δ_PB_ *T* [K]	Δ_PB_ *h* [J kg^−1^]	Δ*h*_st_/Δ*h*_th_ ^a)^	Δ_PB_ *s* [J kg^−1 ^K^−1^]^b)^
225	648.73	2.37	390.06	38.87	164.84
230	650.62	5.83	484.03	19.08	83.03
250	658.04	22.20	688.10	6.50	30.99
270	665.11	39.38	811.88	3.99	20.62
290	671.84	57.03	912.02	2.87	15.99

^a)^ Calculated from equation 24 in ref. [19];

^b)^ Calculated from equation 3.16 in ref. [46] which assumes that *T*_PB_ is the transition temperature in phase equilibrium.

Moreover, while the enthalpy of transition is more than doubled when the pressure increases from 225 to 290 bar, the ratio between structural and thermal contributions of PB decreases by a factor close to 14, and the estimated entropy of transition^[^ ^46^ ^]^ decreases from 165 to 16 J kg^−1^ K^−1^.

**Figure 3:**
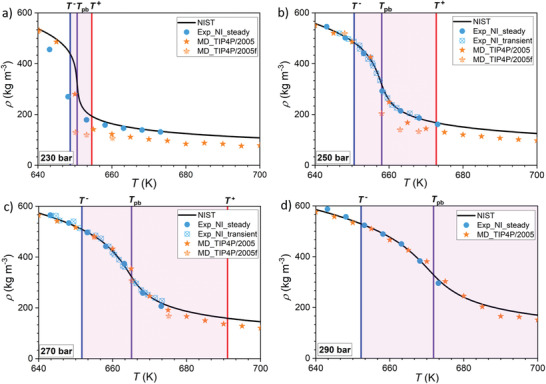
Water density variation versus temperature along different isobars; the symbols represent the density values obtained in this study by neutron imaging (blue) and by MD (orange); the solid lines are plotted for comparison and are based on the NIST reference data,^[^ ^45^ ^]^ the light violet square indicates the PB temperature interval determined as illustrated in Figure 2; the vertical violet line indicates the PB temperature *T*_PB_ corresponding to the maximum of heat capacity; the *T*_PB_ increases and there is a more gentle density decay as the pressure increases.

**Figure** **3** presents the water density drop during isobaric heating in the PB temperature interval at a) 230, b) 250, c) 270, and d) 290 bar. The symbols represent the density values obtained in this study by neutron imaging technique (light blue) and by MD simulations (orange). The experimental density values were calculated from the neutron images recorded in transient and steady‐state conditions (see the Experimental Section and the Supporting Information). To determine the water density by MD, we used TIP4P/2005 rigid^[^ ^47^ ^]^ and flexible^[^ ^48^ ^]^ water models. Details of how we obtain the water density values in this study are presented in the Experimental Section. Additionally, Figure 3 presents the density values reported in the NIST database.^[^ ^45^ ^]^ It is a good agreement between the values obtained in this study with the NIST reference values, although the deviation of our values from NIST values are higher at pressures near the critical one (at 230 bar). Furthermore, Figure 3 shows that increasing the temperature decreases the density of water with the same slope comparing our results with the NIST data. The scH_2_O density decreases at constant pressure from around 600 kg m^−3^ to less than 150 kg m^−3^ when increasing the temperature within the PB temperature interval, and the drop in density by crossing the Widom line at *T*_PB_ becomes flatter with increasing pressure.

**Figure 4:**
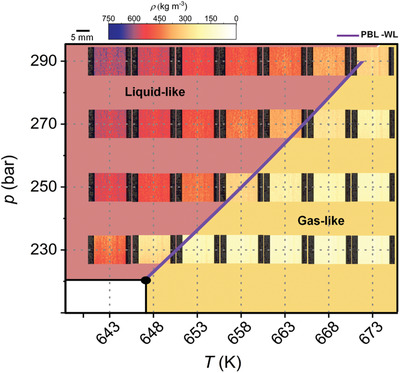
Water phase diagram across the PB–Widom line in *p*–*T* space represented in density images obtained by neutron radiography of the scH_2_O reactor.

**Figure** **4** presents the water phase diagram in pressure–temperature space, in which each state point (*p*, *T*) is represented by a color‐scaled density image. It can be visualized that the PB–Widom line divides the scH_2_O states space in two regions, with LL densities (reddish) above and GL densities (yellowish) below the line. Along the PB–Widom line, the water density has values around *ρ*_cr_  =  322 kg m^−3^. The pale orange color of our density images indicates this observation.

### 2.2 Microscopic View of the Transition

**Figure 5:**
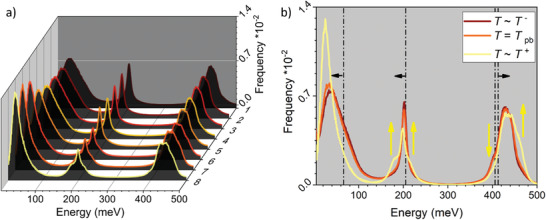
Vibrational spectra of water at different state points calculated by MD using TIP4P/2005f water model. It shows the general trend of a) spectra change and b) the variation at 250 bar when the system evolves from LL to GL states; black dashed lines in (b) indicates the energy zones for the libration, bending, and stretching modes, respectively, as reported for the vibrational spectra of water at ambient conditions;^[^ ^49^ ^]^ yellow arrows in (b) point out the differences between water spectra computed at *T* ^−^, *T*_PB_, and *T* ^+^. It is evident that the red shift is for the libration and bending modes and the blue shift is for the stretching modes relative to the water spectra at ambient conditions. Important is to notice the differences between vibrational spectra for LL and GL, below and above *T*_PB_, respectively.

To obtain structural information, we calculated using MD the water vibrational spectra (**Figure** **5**) for eight state points in the supercritical region close to the critical point. The exact location of these state points on the water phase diagram is presented in Figure S1 in the Supporting Information, and **Table** **2** indicates the corresponding values of pressure and temperature. Figure 5a presents the general trend of the vibrational spectra changes during the phase transition from LL to GL water and Figure 5b shows the vibrational spectra for the state points nos. 2, 4, and 7, corresponding to water at *T* ^−^, *T*_PB_, and *T* ^+^, respectively, on the 250 bar isobar. The dashed gray lines in Figure 5b indicate the energy regions for the libration^[^ ^48^ ^]^ (<100 meV) and the normal vibrational modes of water, one bending mode appearing at ≈200 meV and two stretching modes (symmetric and asymmetric) in the 400 meV energy region^[^ ^49^ ^]^ of the liquid water vibrational spectra (see Figure S2, Supporting Information).

**Table 2:** Values for the water molecule coordination numbers reflecting the changes in the hydrogen bonds network; it includes also the density values reported in the NIST database,^[^ ^45^ ^]^ obtained by neutron imaging and by MD in this study; the state points on the PB–Widom line (4, 5, and 6) are highlighted in bold

SP no.	*T* [K]	*p* [bar]	*ρ*_NIST_ [kg m^−3^]	*ρ*_NI_ [kg m^−3^]	*ρ*_MD_ [kg m^−3^]	〈*n*_HB_〉	CN	Fractions in % of H_2_O molecules with CN = 0, 1, 2, 3, 4, 5, 6
1	640	230	536.8	–	536.8	1.01	2.70	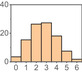
2	648	250	505.5	501.85	525.2	1.05	2.68	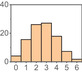
3	655	270	480.95	–	478.6	0.98	2.57	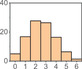
**4**	658	250	312.8	292.3	312.2	0.73	1.88	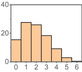
**5**	665	270	321.4	–	268.3	0.74	1.82	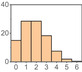
**6**	650	230	377.6	–	137.1^a)^	0.46	1.19	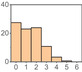
7	668	250	184.1	186.0	133.8	0.35	0.91	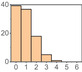
8	660	230	163.2	–	107.1	0.38	0.95	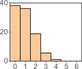

^a)^ For this state point, the TIP4P/2005f interatomic potential is on the sharp density drop.

Comparing the vibrational spectra of water at supercritical and ambient conditions, the blue shift (toward lower energies) of the libration and bending modes, and the red shift (moved to higher energies) of the stretching modes are evident. These shifts are pointed out by black arrows in Figure 5b. More to notice in Figure 5 is that, as the water goes from LL to GL state, the position of the libration band shifts to even lower energy, the peak having higher frequency with narrow distribution. On the contrary, the peaks corresponding to bending and stretching vibration modes have lower frequency and show broader distribution when the system evolves from LL to GL water. The yellow arrows in Figure 5b point out the changes recorded for the bending and stretching vibration modes before and after *T*_PB_. It is evident in the spectrum of GL phase (after the *T*_PB_) that three more shoulders at around 180, 220, and 460 meV appear, and the symmetric stretching band at ≈400 meV disappears. The differences between spectra computed when scH_2_O goes from LL to GL states should be correlated with the structural changes in the hydrogen bonds network. It is reported that the vibrational modes of water are sensitive to the number of hydrogen bonds per water molecule (〈*n*_HB_〉) reflected by changes into the local environment determined by the water molecule coordination number (CN).^[^ ^29^, ^42^, ^48^, ^50^ ^]^ We calculated these values for the eight points under consideration and present in Table 2. The highlighted state points nos. 4, 5, and 6 are on the PB–Widom line (see Figure S1, Supporting Information). First to notice in Table 2 is that above the PB–Widom line, the 〈*n*_HB_〉 has values close to 1 (and CN > 2.5), while below the PB–Widom line the 〈*n*_HB_〉 decreases to around 0.4 (CN ∼0.9). This observation explains the merging of the librations symmetries into a single band,^[^ ^48^ ^]^ the shift of the libration peak location to smaller energies and its increase in the relative frequency intensity. Moreover, the decrease in hydrogen bonding index when the system crosses the PB–Widom line, it explains the shift of the bending peak location to smaller energies and its frequency decreases, suggesting that the HOH angle is less vibrating. More to observe in Table 2 is that, in the transition from LL to GL, the fraction of monomers (isolated water molecules) gradually increases from 3.53% to 38.2% due to the decay of chain associates, the fraction of dimers more than doubles (from 15.4% to 36.2%), while the fraction of trimers decreases from 26.4% to 18.8%, and the fractions of water tetramers reduce drastically from 27.3% to 5.62%. These variations in the local water molecule environment induced by the hydrogen bonding is in agreement with the shifting of the normal modes of stretching toward higher energies, also observed in other studies.^[^ ^29^ ^]^ It is evident in Table 2 that the distribution of water oligomers changes from mostly trimers and tetramers (CN  =  2 and 3) above the PB–Widom line to monomers and dimers (CN  =  0 and 1) frequently below the transition line. The density effect on the vibrational spectra indicates that low‐density water in the supercritical region is considered to be from 10 to 40 kg m^−3^, with mostly monomers^[^ ^29^, ^36^ ^]^ and a certain shape of the OH stretching vibrational profile. There is a density threshold at around 40–50 kg m^−3^ from which dimers occur.^[^ ^29^ ^]^ In the medium‐density region, which is from 100 to 400 kg m^−3^, the density range for the PB, mostly trimers are likely to be present,^[^ ^29^ ^]^ with a doublet profile for the OH stretching mode. However, in our case, the evolution of the shape of the infrared profiles shows the transition from tetramers and trimers to mostly dimers and monomers when crossing the PB–Widom line.

## 3 Discussion

We analyzed the thermodynamics and the structural dynamics of water phase change from LL to GL states in the supercritical region where the PB can be detected (Figure 1). For instance, during isobaric heating of water at constant pressure of 250 bar, the enthalpy of the PB transition is 688 J kg^−1^, while the water density decreases by a factor of close to three. Moreover, our thermodynamic analysis shows that the system needs ≈6.5 times more energy at 250 bars to overcome molecular attractions during LL to GL water structural changes than to increase the temperature of water by 22 K during the PB transition (Figure 2, Table 1 and Figure 3). In addition, our color‐scaled water density images show evident change in color from LL (reddish) to GL (yellowish) densities when the system crosses the PB–Widom line (Figure 4). All these macroscopic changes practically reflect the microscopic variation of water structure, evidenced in this study by the evolution of the shape of the water IR profiles associated with changes in the hydrogen bonds network (Figure 5 and Table 2).

There is a change of symmetry in the first neighboring shell of molecules when water changes its state from liquid at ambient condition to the supercritical region. The tetrahedral structure typical of liquid water at room temperature is substituted in supercritical water by chains of hydrogen‐bonded molecules allowing cavities.^[^ ^35^ ^]^ When the probability of finding a four‐bonded molecule is below the percolation threshold,^[^ ^27^ ^]^ reported to be at an average number of hydrogen bonds per H_2_O molecule of 1.6,^[^ ^51^ ^]^ the tetrahedral spatial arrangement becomes unlikely.

**Figure 6:**
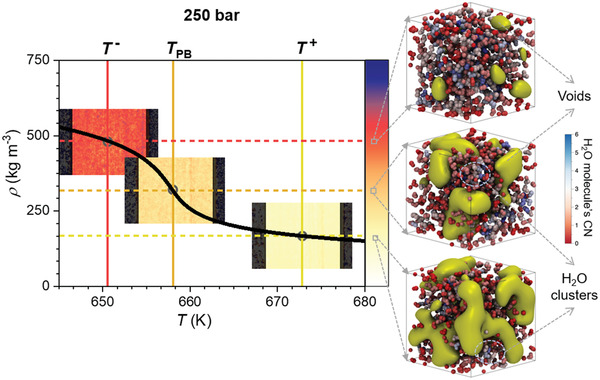
Correlation between density drop and structural changes associated with the PB; it presents the density variation with the temperature at 250 bar, the colored‐scaled density images recorded by neutron imaging, and the corresponding snapshots of the MD computational supercell.

The correlation between density variation associated with the PB (thermodynamics) and structural changes revealing the dynamics of the transition is illustrated in **Figure** **6** for the 250 bar isobar. It presents the density images recorded at temperatures close to *T* ^−^, *T*_PB_, and *T* ^+^, respectively, while showing the drop of water density from LL (around 500 kg m^−3^) to GL (around 150 kg m^−3^) values, and having the value close to the critical density at *T*_PB_. On the right, we present the snapshots of the MD computational supercell at the same temperatures of the recorded density images. They show the water molecules and void isosurfaces representing those points in space, to which all water molecules exhibit a distance higher than 3 Å.^[^ ^52^ ^]^ For the 630–690 K temperature range, in the Supporting Information we present the video (Movie S1) including large number as such snapshots. At temperatures close to *T* ^−^ in LL state, the water molecules are homogenously distributed. The PB transition starts, and voids are formed and increase in size as the temperature increases (green “bubbles” in the structural snapshots). There is a temperature (water density) at which the merging of voids practically isolates water molecules clusters. These are metastable aggregates with larger density than the ideal gas.^[^ ^52^ ^]^ This is evident in our structural images for temperatures at and higher than *T*_PB_.

## 4 Conclusion

This work presented the first quantitative analysis of the thermodynamics and the structural dynamics of the PB phase transition from LL to GL states of water, and show how the macroscopic variation of water properties reflects the microscopic changes in the water structure in the pressure–temperature space of the supercritical region delimited by *p*/*p*_cr_  =  3 and *T*/*T*_cr_  =  1.5. Based on the PB theory, the phase transition boundaries were determined, and more the thermodynamic significance of the heat capacity‐based Widom line, the demarcation line between the two scH_2_O phases, was given. The thermodynamic analysis indicated that, at constant pressure near the critical point, the system needs roughly 40 times more energy to change its structure from LL to GL phase than to increase its temperature by 2.4 K in the PB transitional region. In the same region, it was observed by in situ neutron imaging that the water density drops by a factor of three, regardless of pressure, having values close to the critical density at the PB–Widom line.

The MD simulations showed that the structure of LL water above the PB–Widom line consists mainly by trimer and tetramer clusters, while below the PB–Widom line, the water in GL state is mostly formed by dimers and monomers. More, it has been shown that the PB phenomenon is similar to the subcritical water boiling. The process is initiated by the formation of small vacuum cavities, which increase in size, merge together, and isolate clusters of water molecules with density higher than the ideal gas.

This fundamental knowledge allows to establish the structure–properties relationship necessary to finely tune the thermodynamic, transport, and solvent properties of scH_2_O.

## 5 Experimental Section

##### Thermodynamic Analysis

Thermodynamic analysis was performed on reference data for water, as provided by NIST^[^ ^45^ ^]^ using the linear enthalpy model.^[^ ^19^, ^20^ ^]^ In this model, the enthalpy–temperature relation was given by linear approximations in the liquid and gaseous limits, respectively, and at PB. These functions were of the form $h\;\left(T\right)=c_{p}^{k}\;\;\left(T-T^{k}\right)+h_{0}^{k}$, where *k* denotes the reference state, i.e., liquid L, gaseous G, and PB. Reference parameters were obtained as $h_{0}^{G},\;\;c_{p,0}^{G}=\;f\left(p\;=\;0\;Pa,\;T\;=T_{\mathrm{cr}}\;\;\right);\;h_{0}^{L},\;c_{p}^{L}=\;f\left(p\;=p_{\mathrm{cr}}\;,\;T\;=\;0.5T_{\mathrm{cr}}\right);\;h_{0}^{\mathrm{PB}},\;c_{p}^{\mathrm{PB}}=\;f\left(\mathrm{PB}\right)$, where the PB state was characterized as having a maximum specific isobaric heat capacity for an isobaric temperature scan. The transition limit temperatures were defined by the intersections of the respective linear enthalpies, i.e., $T^{-}\;\left(p\right)=\;\left[T|\;h_{0}^{L}\left(T\right)\;=h_{0}^{\mathrm{PB}}\;\left(T,p\right)\right]$, and $T^{+}\;\left(p\right)=\;\left[T|\;h_{0}^{G}\left(T\right)\;=h_{0}^{\mathrm{PB}}\;\left(T,p\right)\right]$. The procedure with the enthalpy functions is illustrated in Figure 2.

##### Neutron Imaging

For the in situ neutron imaging experiments, the dedicated setup called NISA (**N**eutron **I**maging **S**upercritical‐water **A**nalysis) was used. The schematic representation was presented elsewhere.^[^ ^23^ ^]^ Briefly, the setup was consisted of 12 mm inside diameter continuous flow tubular reactor (SITEC, Switzerland), heating elements (preheater and aluminium block heater), heat exchanger, back pressure regulator (TESCOM, USA), high precision liquid chromatography pumps (Knauer, Germany), balances (Sartorius, Germany), temperature, and pressure sensors. The material of the reactor (Zircaloy‐4, grade R60804) was chosen based on the particularly harsh experimental conditions applied (high temperature and high pressure) and its transparency to neutrons. The setup was remotely controlled via graphical operator terminal and parameters such as temperature, pressure, and mass flow rate, were monitored on‐line and acquired via DAQ application written in LabView (Computer Power SRL, Romania). In the present work, neutron imaging measurements of pure water, in both steady‐state and transient conditions, were performed in the thermodynamic conditions in the vicinity of the critical point of water. The water flow was plugged into the tubular reactor with 5 mL min^−1^ rate. For each experimental point, the steady‐state conditions were kept for 10 min prior to the neutron measurements. The temperature was increased with a step of 5 K along four isobars at 230, 250, 270, and 290 bar, respectively.

The neutron imaging was performed at the NEUTRA beam line^[^ ^53^ ^]^ (SINQ source, Paul Scherrer Institute), which had a thermal neutron energy spectrum with a maximal intensity at 25 meV. The sample was placed at the measurement position no. 2, which was 7 m away from the source aperture, and the sample–detector distance was ≈100 mm. This experiment geometry (shown in Figure S5, Supporting Information) with the NEUTRA source aperture of 20 mm resulted in a geometrical blurring of 0.3 mm. The neutrons were captured and converted to visible light with a 50 µm thick 6LiF/ZnS scintillator screen and the resulting image was recorded by a CMOS camera (Andor Neo sCMOS, 2160 × 2560 pixels). With the used optical setup, the resulting pixel size was 47 µm. Each image was recorded with an exposure time of 30 s. Details of the imaging processing procedure to obtain the water density images shown in Figures 4 and 6, are presented in the Supporting Information.

##### MD

Simulations of scH_2_O at different thermodynamic conditions (state points) were performed using GROMACS v.2016‐4 code.^[^ ^54^ ^]^ The vibrational spectra (DoS) were calculated using the flexible TIP4P/2005f^[^ ^48^ ^]^ interatomic potential. The supercell was consisting of 2048 water molecules in a cubic box with periodic boundary conditions. Three different isobars (230, 250, and 270 bar) were examined in the temperature range of 648–668 K in order to determine the effect of the LL to GL transition in the vibrational spectra. The equilibrium runs were performed in the isothermal–isobaric (NPT) ensemble and the sampling runs were performed in the canonical (NVT) and microcanonical (NVE) ensemble. The equations of motion were integrated using the leapfrog algorithm with a time‐step of 0.1 fs (in order to capture the fast‐internal vibrations)^[^ ^55^ ^]^ for a total sampling time of 100 ps. The particle‐mesh Ewald method was used to evaluate the long‐range electrostatic interactions with a cut‐off of 1.4 nm. An offset both in temperature and pressure was applied in order to reproduce the experimental critical point (temperature and pressure).^[^ ^34^ ^]^ In the CNs calculation, a cut off of 3.5 Å was used, and for the hydrogen bonds, a geometric criterion based on the oxygen–oxygen distance (*d*_OO_ < 3.5 Å)^[^ ^56^ ^]^ and hydrogen–oxygen–hydrogen angle (HOH < 30°) was used.^[^ ^34^ ^]^

## Conflict of Interest

The authors declare no conflict of interest.

## Supporting information

Supporting Information

Supplemental Movie 1
