# Supplementary material for: Thermodynamics and Dynamics of Supercritical Water Pseudo‐Boiling
Source: Adv Sci (Weinh). 2020 Dec 16;8(3):2002312. doi: 10.1002/advs.202002312 (PMC7856905; doi:10.1002/advs.202002312)
Supplement: Supplementary file 1 — Supporting Information [file ADVS-8-2002312-s001.pdf]

© 2020 Wiley-VCH GmbH

Supporting Information

for *Adv. Sci.,* DOI: 10.1002/advs.202002312

Thermodynamics and Dynamics of Supercritical Water Pseudo-Boiling

Florentina Maxim, Konstantinos Karalis, Pierre Boillat, Daniel T. Banuti, Jose Ignacio Marquez Damian, Bojan Niceno, and Christian Ludwig**

Supporting Information

Thermodynamics and Dynamics of Supercritical Water *Pseudo*-Boiling

Florentina Maxim, Konstantinos Karalis, Pierre Boillat, Daniel T. Banuti, Jose Ignacio Marquez Damian, Bojan Niceno, and Christian Ludwig**

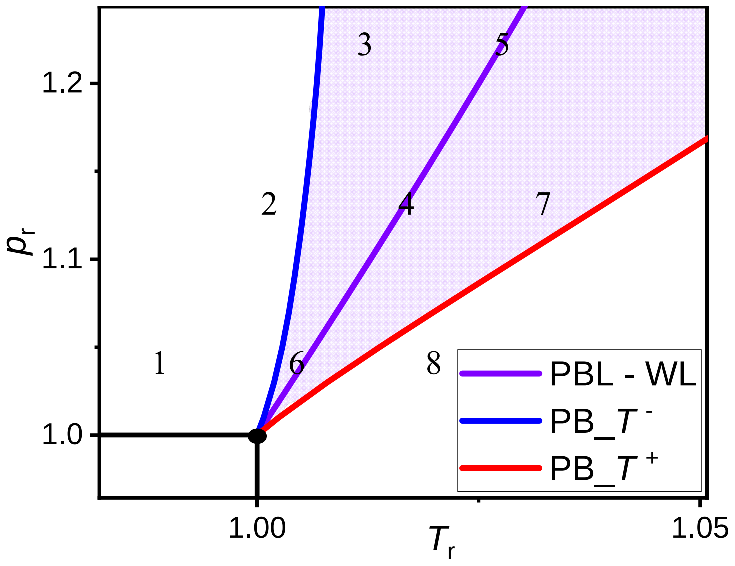

Figure S1. Water phase diagram in p_r-T_r space with the indication of the 8 state points at which refers to the Figure 5.

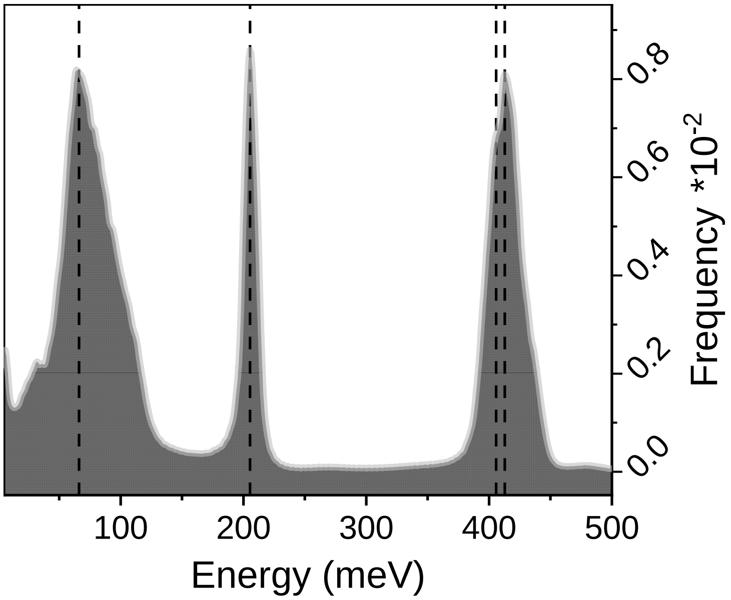

Figure S2. Vibrational spectra for liquid water at ambient conditions.

Neutron images processing procedure: It is illustrated in **Figure** and was conducted as follows. The raw images were filtered to remove the white spots corresponding to gamma rays hitting the detector, corrected for the background contributions (camera offset, scattered neutrons) and for the beam intensity fluctuations, and divided pixel-wise by the image of the dry reactor to remove the contributions from the attenuation of the reactor itself. The corresponding relative transmission image ($\frac{I}{I_0}$) was converted to an equivalent water thickness image (δ) using the following equation:

$$\delta = -k_1 \cdot \ln\left(\frac{I}{I_0}\right) - k_2 \cdot \left[\ln\left(\frac{I}{I_0}\right)\right]^2 - k_3 \cdot \left[\ln\left(\frac{I}{I_0}\right)\right]^3 \qquad \text{Equation S1}$$

Where the values of the parameters ($k_1 = 2.738$ mm, $k_2 = 0.103$ mm and $k_3 = -0.002$ mm for supercritical water and $k_1 = 2.795$ mm, $k_2 = 0.0066$ mm and $k_3 = 0.0009$ mm for liquid water) were determined based on the known neutron energy spectrum and energy dependent neutron cross section of water. The non-linear relation between δ and $ln\left(\frac{I}{I_0}\right)$ stems from beam hardening (a reduction of the effective neutron cross section with increasing material thickness due to the energy dependency of the neutron cross section). Using the known Lambert-Beer relation for each individual wavelength, the attenuation of the integral beam intensity as a function of water thickness is given by equation S2, where $I(\lambda)$ is the wavelength dependent beam intensity, $eff(\lambda)$ is the wavelength dependent detector efficiency, $\sigma(\lambda)$ is the wavelength dependent water cross section per water molecule and N is the number of water molecules per unit volume.

$$\frac{I(\delta)}{I_0} = \frac{\int I(\lambda) \cdot eff(\lambda) \cdot e^{-\sigma(\lambda) \cdot N \cdot \delta} \cdot d\lambda}{\int I(\lambda) \cdot eff(\lambda) \cdot d\lambda} \qquad \text{Equation S2}$$

The wavelength dependent cross sections used for this calculation are shown in Figure S4. For liquid water, the known values from the EXFOR experimental cross sections database were used. For supercritical water, we performed energy-selective measurements at the ICON beam

line of PSI [1]. Because equation S2 is not solvable analytically for δ, the ratio $\frac{I}{I_0}$ was computed for a series of thickness values covering the range of interest, and a 3rd order polynomial fit (resulting in the parameters k_1, k_2 and k_3) was used as an approximation of the relation between $ln\left(\frac{I}{I_0}\right)$ and δ. The equivalent thickness image was finally converted to a density image by dividing pixel-wise the thickness with the measured thickness in a reference condition where the reactor was filled with liquid water. For display purposes, the density images were converted in false colour with blue representing the liquid water density, red representing the density of LL supercritical water (~600 kg m^{-3}) and yellow representing the density of GL supercritical water (below 200 kg m^{-3}).

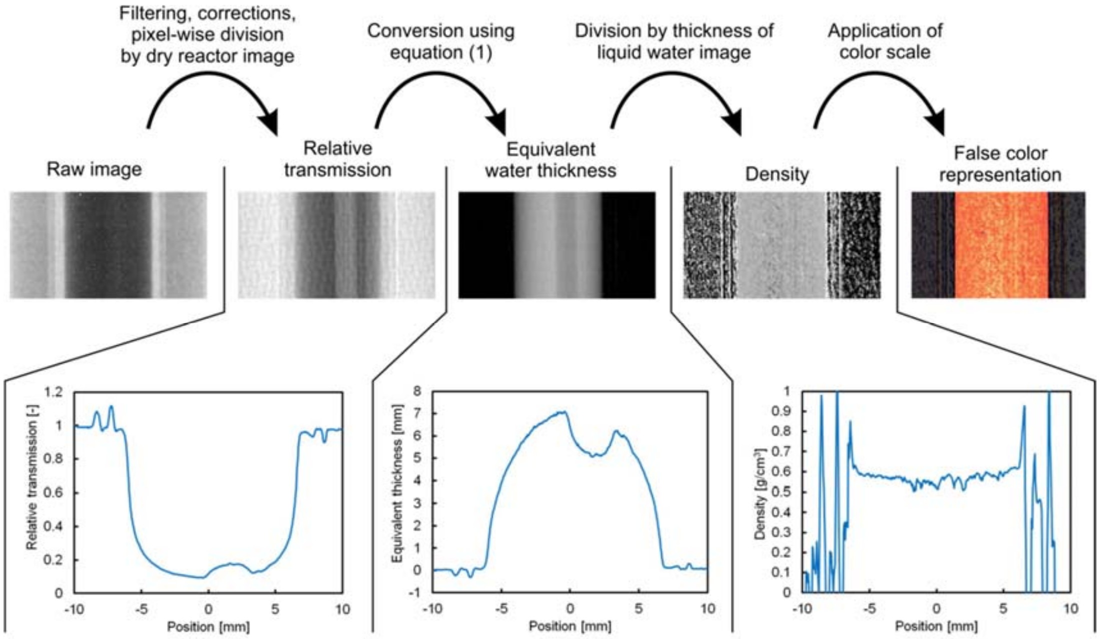

Figure S3. Illustration of the image data processing procedure; the raw image is filtered, corrected and pixel-wise divided by dry reactor image; the relative transmission image is converted to equivalent water thickness using Equation S1; the density images is obtained dividing the thickness to the liquid water image; for display purpose a false colour scale is applied.

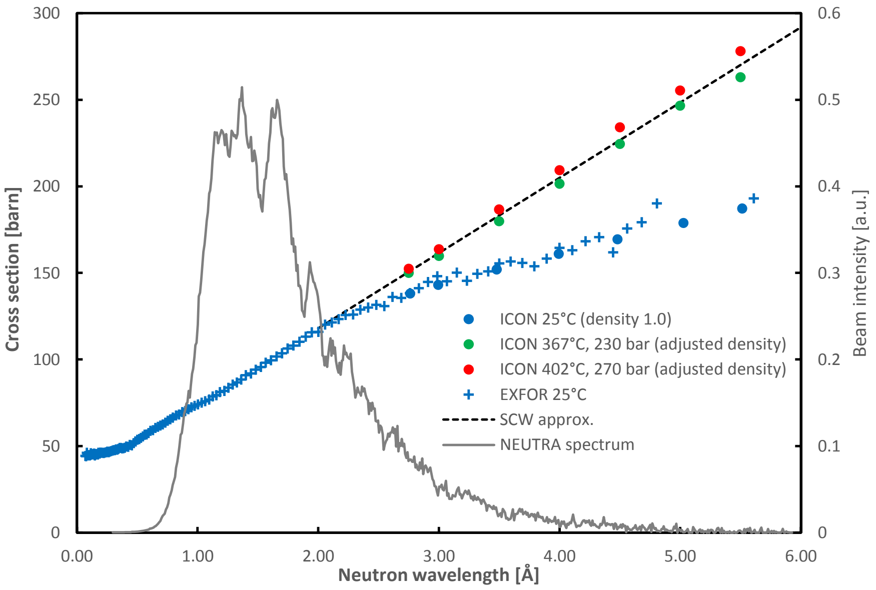

Figure S4. Experimental energy-dependent cross sections per molecule for room temperature liquid water and supercritical water (two different conditions shown). The density of supercritical water was not assumed to be known *a priori*, but adjusted so that the extrapolation of the cross-section fits the cross section of liquid water at a wavelength of 2Å (this is a reasonable assumption given that the cross section at this low wavelength only marginally changes with temperature[2]). The temperature dependence of the water cross-sections only marginally affects measurements at NEUTRA, because the intensity peak at this beam line is between 1Å and 2Å. Note: 1 [barn] = 10^{-24} [cm^{-2}]

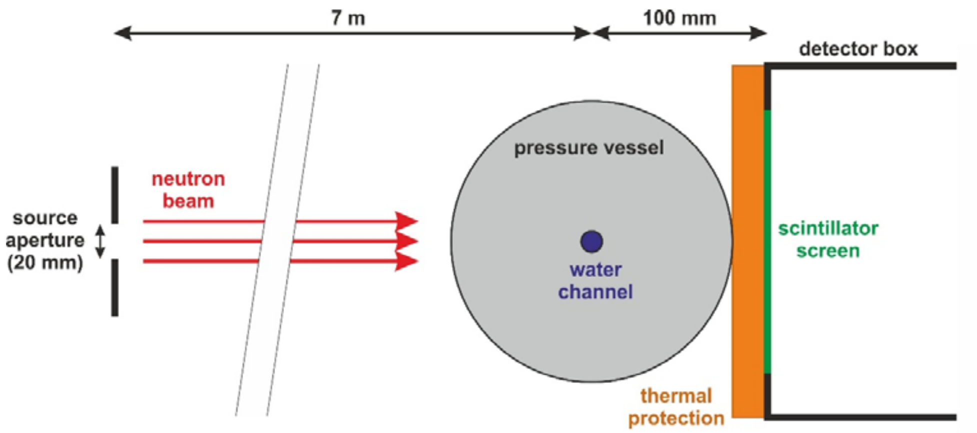

Figure S5. Basic description of the experiment geometry.

References:

1. Kaestner, A.P., Hartmann, S., Kühne, G., Frei, G., Grünzweig, C., Josic, L., Schmid, F., and Lehmann, E.H. (2011) The ICON beamline A facility for cold neutron imaging at SINQ. Nucl. Instruments Methods Phys. Res. Sect. A Accel. Spectrometers, Detect. Assoc. Equip., 659 (1), 387–393.

2. Marquez Damian, J.I., Dawidowski, J., Granada, R.J., Cantargi, F., Romanelli, G., Helman, C., Krzystyniak, M., Skoro, G., and Roubtsov, D. (2020) Experimental validation of the temperature behavior of the ENDF/B-VIII.0 thermal scattering kernel for light water. EPJ Web Conf., 239, 14001.
